# *REV-ERB ALPHA* Polymorphism Is Associated with Obesity in the Spanish Obese Male Population

**DOI:** 10.1371/journal.pone.0104065

**Published:** 2014-08-04

**Authors:** Elena G. Ruano, Silvia Canivell, Elaine Vieira

**Affiliations:** 1 CIBER de Diabetes y Enfermedades Metabólicas Asociadas (CIBERDEM), Barcelona, Spain; 2 Diabetes and Obesity Research Laboratory, IDIBAPS, Barcelona, Spain; Universidad Miguel Hernández de Elche, Spain

## Abstract

*REV-ERB ALPHA* has been shown to link metabolism with circadian rhythms. We aimed to identify new polymorphisms in the promoter of *REV-ERB ALPHA* and tested whether these polymorphisms could be associated with obesity in the Spanish population. Of the 1197 subjects included in our study, 779 were obese (BMI 34.38±3.1 kg/m^2^) and 418 lean (BMI 23.27±1.5 kg/m^2^). In the obese group, 469 of the 779 had type 2 diabetes. Genomic DNA from all the subjects was obtained from peripheral blood cells and the genotyping in the *REV-ERB ALPHA* promoter was analyzed by High Resolution Melting. We found six polymorphisms in the *REV-ERB ALPHA* promoter and identified rs939347 as a SNP with the highest frequency in the total population. We did not find any association between rs939347 and type 2 diabetes (p = 0.101), but rs939347 was associated with obesity (p = 0.036) with the genotype AA exhibiting higher frequency in the obese (5.2% in total obese vs 2.4% in lean). This association was found only in men (p = 0.031; 6.5% AA-carriers in obese men vs 1.9% AA-carriers in lean men), with no association found in the female population (p = 0.505; 4.4% AA-carriers in obese women vs 2.7% AA-carriers in lean women). Our results suggest that the *REV-ERB ALPHA* rs939347 polymorphism could modulate body fat mass in men. The present work supports the role of *REV-ERB ALPHA* in the development of obesity as well as a potential target for the treatment of obesity.

## Introduction

Obesity is considered the epidemic of the 21^st^ century affecting almost 500 millions of people, which represents 11% of the world population [1]. Recently, new possible contributing factors to obesity have been identified. Among them, the disruption of the circadian rhythms has emerged as one of the main risk factors to develop obesity and type 2 diabetes in humans [2], [3] and in mice [4]–[6]. Genetic variations within Clock genes have been associated with obesity and metabolic syndrome [7]–[11].

Single nucleotide polymorphisms (SNPs) in the Clock genes have been extensively studied in recent times. Genetic variations in the *CLOCK* (Circadian Locomotor Output Cycles Kaput) were associated with overweight and obesity, food intake and metabolic syndrome traits [7], [8]. Interestingly, these genetic effects on insulin resistance and obesity may be modulated by the dietary intake of monounsaturated fatty acids and saturated fatty acids [9]. Genetic variations in two haplotypes of the *BMAL1* gene were found to be associated with hypertension and type 2 diabetes [12]. The nuclear receptor *REV-ERB ALPHA* (also known NR1D1) is an integral component of the circadian clock machinery [13], [14] and was suggested to play an important role in determining the correct phase of *BMAL1-CLOCK* target genes [13], [15]. In addition to regulating the clock machinery, *REV-ERB ALPHA* was also shown to link circadian rhythms with metabolism. *REV-ERB ALPHA* was found to regulate inflammation [16], [17] glucose metabolism [18]–[20], and oxidative capacity in skeletal muscle [21] and plays an important role in the regulation of adipogenesis [22], [23]. Studies on genetic variations of *REV-ERB ALPHA* gene and obesity have shown that the rs2071427 polymorphism modulates body fat mass in both adult and young people [10]. More recently, another polymorphism in the *REV-ERB ALPHA* gene rs2314339 was associated with obesity in two cohorts from Mediterranean and North American population [11]. However, these genetic variations were found only in introns of the *REV-ERB ALPHA* gene.

Here, we identified SNP rs939347 located in the promoter region of the *REV-ERB ALPHA* gene. This polymorphism was associated with obesity only in the Spanish male population, indicating a gender-specific role in the genetic variation of *REV-ERB ALPHA* in the development of obesity. These results support the relevant role of REV-ERB ALPHA in human obesity and provide further evidence for sexual dimorphisms in the interaction of the circadian clock.

## Research Design and Methods

### Subjects

A cross-sectional study with a total of 1197 subjects from the Hospital Clinic-IDIBAPS Biobank (Barcelona; Spain) [24] and the National DNA Bank (Salamanca; Spain) [25] were studied. Among the total population 779 were obese (BMI 34.38±3.1 kg/m^2^) and 418 lean (BMI 23.27±1.5 kg/m^2^). In the obese group, 469 of 779 subjects had type 2 diabetes according to American Association of Diabetes guidelines [26]. The clinical characteristics of the subjects are described in the Table 1. To summarize, all the subjects were from Spain, with ages ranging from 62.7±8.9 in lean subjects and 64.3±9.0 in the total obese subjects. The proportion male/female was similar in both groups (41.4% men and 58.6% women in the obese population, and 37.4% men and 62.6% women in the lean group). Subjects with history of malignant disease or a major chronic illness were excluded from the study. This study was approved by the Ethics Committee of the Hospital Clinic-IDIBAPS (Barcelona) and by the Scientific and Ethics Committee of the Spanish National DNA Bank, University of Salamanca, Spain. All human subjects provided written informed consent prior to participating in the study.

**Table 1:** Clinical characteristics of the subjects.

	*Lean*	*OB*	*T2D*	*TOTAL*	*P1*	*P2*	*P3*	*P4*
*N*	*418*	*310*	*469*	*779*				
***Age***	62.7±8.9	62.1±8.8	65.8±8.9	64.33±9.0	0.003	NS	0.001	0.001
***Male %***	37.4% (157)	36.7% (114)	44.5% (209)	41.4% (323)	NS	NS	0.04	0.03
***BMI*** ** (kg/m^2^)**	23.27±1.5	34.8±3.0	34.1±3.8	34.38±3.1	0.000	0.000	0.000	0.01
***Waist circumf. (cm)***	82.81±8.1	110.3±11.6	111.6±9.9	111.12±10.6	0.000	0.000	0.000	NS
***Glucose (mg/dL)***	87.6±11.8	103.5±18.9	162.1±58.8	140.47±55.6	0.000	0.000	0.000	0.000
***Hb1%c***	ND	5.4±0.6	7.6±1.5	7.03±1.6				0.000
***Cholesterol (mg/dL)***	202.4±55.1	202.2±41.6	182.1±40.2	188.91±42.9	0.000	NS	0.000	0.000
***LDL (mg/dL)***	107.48±37.9	127.5±35.7	106.6±32.2	113.54±35.7	NS	0.000	NS	0.000
***HDL (mg/dL)***	57.93±18.5	50.2±14.3	46.3±16.6	48.03±19.9	0.000	0.000	0.000	0.003
***Triglycerides (mg/dL)***	90.78±41.9	137±82.9	158.7±97.1	150.0±92.9	0.000	0.000	0.000	0.004
***C-reactive protein (mg/L)***	1.7±5.1	4.8±6.3	5.0±5.8	4.8±6.0	0.002	0.01	0.009	NS
***Cortisol (ng/mL)***	104.2±54.9	69.7±40.2	79.9±46.8	73.3±42.9	0.000	0.000	0.000	NS
***Melatonin (pg/mL)***	16.1±9.4	9.2±7.9	8.5±6.0	8.8±6.9	0.000	0.000	0.000	NS

P1 =  Lean vs total obese population; P2 =  Lean vs Obese (OB); P3 =  Lean vs type 2 diabetes (T2D); P4 =  OB vs T2D.

### DNA isolation and Serum Samples

Genomic DNA from all subjects was obtained from peripheral blood cells according to the procedures of the Hospital Clinic-IDIBAPS Biobank (Barcelona; Spain) [24] and the National DNA Bank (Salamanca; Spain) [25]. Serum samples were obtained in a fasting state between 8∶00 am and 9∶00 am, processed and preserved at −80°C. This study was approved by Ethics Committee of the Hospital Clinic of Barcelona and by the Biobank Ethics Committee in compliance with all laws and international ethics guidelines outlined in the Declaration of Helsinki. All human subjects provided written, informed consent prior to the study.

### Genotype analysis

The region between −1080 bp and +79 bp of the *REV-ERB ALPHA* promoter was analyzed by High Resolution Melting (HRM) using LightCycler 480 PCR Real-Time (Roche, Basel, Switzerland). The promoter was divided into four PCR fragments (1A, 1B, 1C, 1D) each of ≈300 bp. The primers used are described in the Table S1. The total volume in each reaction was 10 µL, with 0.25 µM of each pair of primers, 1.5 mM of Mg^2+^, 1X of HRM Melting Master (Roche) and 25 ng of DNA. The PCR conditions were as follows: an initial denaturalization at 95°C for 10 minutes followed by 45 cycles of 10 seconds at 95°C, 15 seconds at 58°C (for 1A, 1B and 1D amplicons) or at 55°C (for 1C amplicon) and 12 seconds at 72°C. The HRM cycle was done at 95°C for 1 minute and 40°C for 1 minute followed by melting from 75°C to 95°C with 25 adquisitions/°C and 30 seconds of cooling to 40°C with a ramp of 2.2°C/second. Amplification and melting curves were generated and analyzed by LightCycler480 Gene Scanning Module software version 1.5 (Roche). Those samples whose melting patterns were different from a control sample previously sequenced, were purified using NucleoSpin Gel and PCR Clean Up (Macherey-Nagel, Düren, Germany) following the manufacturer’s instructions. The purified products were sequenced using Big DyeTerminator V3.1 (Applied Biosystems, Inc., Fostercity, CA, USA) in an ABI 377 sequencer (Applied Biosystems) at the DNA Unit of the Hospital Clinic (Barcelona, Spain). The electropherograms were analyzed with Chromas Lite Software 2.1 (www.technelysium.com.au) and compared with the genome browser from the University of California Santa Cruz (http://genome.ucsc.edu/).

### Genotyping of rs2071427

For allelic discrimination of SNP rs2071427 we used a pre-designed Taqman assay (Applied Biosystems). PCR reactions were performed in 96-well plates on the ABI7900HT Fast Real-Time system (Applied Biosystems) using 5 ng of genomic DNA. Genotype was detected with SDS 2.4.1 Software (Applied Biosystems).

### Cortisol and melatonin measurements

Cortisol and melatonin were quantified from serum samples by ELISA (IBL, Hamburg, Germany) according to the manufacturer’s instructions. Standard curve and analysis were performed with Gen5.10 Software (BioTek, Winooski, VT, USA).

### Cloning and directed mutagenesis

The 1670 bp fragment corresponding to the promoter of *REV-ERB ALPHA* was cloned into the pGL3-Basic vector (Promega, Madison, Wisconsin, USA) containing the luciferase reporter gene, using BglII restriction-site. The following primers were used to amplify the promoter: Forward: 5′ACGTCTGACCTTGGGTCGGTCA 3′, Reverse: 5′ CACTAAAGCACCGCAGCACG3′. We inserted the minor allele of rs939347 through directed mutagenesis using QuickChange II Site-Directed Mutagenesis Kit (Stratagene, La Jolla, CA, USA) following the manufacturer’s instructions. The primers used were designed with QuickChange Primer Design (Stratagene). All the constructs were sequenced to confirm their integrity and to detect the presence of mutations.

### Cell lines and luciferase assay

The cell lines HEK-293 and mouse preadipocytes 3T3-L1 were grown in Dubelcco’s Modified Eagle Medium (DMEM) (Lonza, Basel, Switzerland) supplemented with 10% of fetal bovine serum or calf serum and 1% of mix of penicillin and streptomycin at 37% in 5% of CO_2_ atmosphere. The transient transfection was performed at 60% of confluence in 6-well plates using TransFast Transfection reagent (Promega) with a ratio of 2∶1 and 1225 ng of each pGL3 construct, 850 ng of pCMV6-XL4-Flag-PPARγ (kindly provided by Dr. Marcelina Parrizas, from IDIBAPS, Barcelona) and 20 ng of pRL-CMV (kindly provided by Dr. Rosa Gasa from IDIBAPS, Barcelona), following the manufacturer’s instructions. Forty-eight hours after transfection the luciferase activities were measured using Dual Luciferase Assay kit (Promega). The *Firefly* luciferase activity was normalized against the *Renilla* luciferase activity. The assay was repeated three times.

### Statistical analysis and Haplotype analysis

To determine the differences in the clinical data corresponding to lean, obese, type 2 diabetes we performed a one-way ANOVA and unpaired t-test using Graphpad prism 5.0 (www.graphpad.com). To study the genotype and allele distribution in each population we performed a χ^2^ test, using SPSS 18.0 software (SPSS, Chicago, IL, USA). Hardy-Weinberg equilibrium test was carried out as previously. The linkage desequilibrium study was performed using HAPLOVIEW 4.0 (http://www.broad.mit.edu/mpg/haploview/) assuming as linkage desequilibrium an R^2^>0.8. [27] The haplotype study was carried out with Thesias 3.1 which allowed for not only estimating the haplotype frequency but also their associated effects [28]. Logistic regression models were built to study the association of obesity with the AA-genotype from SNP rs939347. In addition, we also performed logistic regression models of having the AA-geneotype as compared to GG- and AG- genotypes in relation to clinical features such as BMI and waist circumference. Stratification by gender was done, as well as adjustment for age and gender using STATA.11 (StataCorpo LP, TX, USA). To test the associations between *REV-ERB ALPHA* rs939347 and obesity-related features such as BMI and waist circumference we performed one-way ANOVA and Mann-Whitney tests. To determine the expression differences in the luciferase assay between the different groups we carried out a one-way ANOVA test. All p-values were 2-tailed with p values <0.05 were considered as significant.

## Results

### Clinical features

The clinical data of the subjects are described in the Table 1. Briefly, the obese and type 2 diabetic subjects were slightly older than the lean subjects and exhibited most common features of obesity and type 2 diabetes: high BMI and waist circumference, elevated plasma glucose values, HbA1c levels, high triglycerides levels, low HDL levels and high levels of inflammation, evident in the elevated levels of C-reactive protein. Morning cortisol and melatonin levels were decreased in the obese and type 2 diabetic subjects as compared to lean subjects.

In addition, we compared the obese and type 2 diabetes subjects, observing that the type 2 diabetes group is older than the obese and have comparatively smaller BMI than the obese subjects. As expected, the type 2 diabetes subjects showed higher fasting glucose levels and higher glycated haemoglobin. There were no differences in morning cortisol and melatonin between the obese and the type 2 diabetes subjects (Table 1).

### Association study of SNPs in the *REV-ERB ALPHA* promoter with obesity and obesity parameters

We first performed a screening in the *REV-ERB ALPHA* promoter in order to find new SNPs that could be associated with obesity and type 2 diabetes. HRM analysis showed six SNPs in the *REV-ERB ALPHA* promoter that are described in the Table S2. All SNPs described in Table S2 have been previously reported in the HapMap project (http://www.ncbi.nlm.nih.gov/projects/SNP/). Among the SNPs in the *REV-ERB ALPHA* promoter, we decided to study SNPs rs939347, which exhibited values (>0.1) in the minor allele frequency analysis and is tagging with rs2071570 [10]. Moreover, we studied the rs2071427, which has been previously described to be associated with BMI in adolescents and adults in European cohorts [10]. We first performed the linkage disequilibrium analysis of both rs939347 and rs2071427 by Haploview 4.0. The analysis confirmed that these two SNPs were not in linkage desequilibrium in our cohort (r-squared = 0.03).

We next checked the association of these SNPs with obesity and type 2 diabetes. The χ^2^ showed no association between rs2071427 with obesity (p = 0.769) and type 2 diabetes (p = 0.232) nor when analyzed by gender (p = 0.476 in men; p = 0.228 in women), (Table 2). We also did not find any association between rs939347 and type 2 diabetes (p = 0.101). Strikingly, rs939347 was found to be associated with obesity (p = 0.036) (Table 2) with the genotype AA exhibiting a higher proportion in the total obese population (5.2% in total obese population vs 2.4% in lean). This association was more relevant in men (p = 0.031; 6.5% AA-carriers in obese men vs 1.9% AA-carriers in lean men) than in women (p = 0.505; 4.4% AA-carriers in obese women vs 2.7% AA-carriers in lean women) (Table 2). Logistic regression analysis confirmed the association between the AA genotype of rs939347 with BMI values between 30 and 35 (p = 0.019; OR = 2.40 [1.14–5.04]), and waist circumference (p = 0.014; OR = 1.03 [1.01–1.05]) (Table 3). The AA genotype association of rs939347 with BMI was found to be present in the male (p = 0.039) but not in the female population (Table 3).

**Table 2:** Genotype distribution of rs939347 among lean, obese and T2D subjects by gender.

SNP	Lean			Obese			T2D			P1	P2	P3	P4
rs939347	Men	Women	Total	Men	Women	Total	Men	Women	Total				
GG	93	168	261	80	124	204	123	167	290	0.036	0.023	0.101	0.390
AG	61	85	146	25	62	87	72	82	154				
AA	3	7	10	8	9	17	13	11	24				
Total	155	257	417	113	195	308	208	260	468				
rs2071427													
CC	85	150	235	81	110	191	117	145	262	0.769	0.280	0.996	0.232
CT	61	96	157	27	71	98	81	95	176				
TT	11	15	26	5	14	19	10	19	29				
Total	157	261	418	113	195	308	208	259	467				

P1 =  Lean vs total obese population; P2 =  Controls vs Obese (OB); P3 =  Controls vs type 2 diabetes (T2D); P4 =  Ob vs T2D.

**Table 3:** Association of risk genotype AA vs AG+GG in rs939347 with study variables.

	OR [95%CI]	P Value	AdjustedP value*	Adjusted P value*in men	Adjusted P value*in women
***BMI*** (n = 1 190)	1.05 [1.00–1.10]	0.062	0.053	0.039	0.554
***BMI≤30*** (n = 415)	Reference group				
***30<BMI<35*** (n = 466)	2.40 [1.14–5.04]	0.021	0.019	0.078	0.133
***BMI≥35*** (n = 309)	2.07 [0.92–4.67]	0.080	0.071	0.051	0.662
***Waist circumference*** (n = 888)	1.03 [1.01–1.05]	0.014	0.010	0.066	0.094
***Waist circumference at*** risk **(n = 888)	2.35 [0.83–6.69]	0.108	0.081	0.105	0.460

Results of logistic regresion models of having the genotype AA versus having the genotype AG and/or the genotype GG in the SNP 939347 by increasing levels of the aforementioned variables. *Multivariate models were creating adjusting by age and gender. **if waist circumference >94 cm in men and >80 cm in women, according to EGIR criteria (1999).

The association between rs939347 and the risk of obesity analyzed by logistic regression showed that the AA carriers exhibited the highest risk for obesity as compared to GG carriers (p value = 0.024; OR = 2.28 (1.12–4.64) and as compared to AG carriers (p value = 0.015; OR = 2.46 (1.19–5.06) (Table 4). Importantly, these results were also confirmed in the male population (AA vs AG p value = 0.035; OR = 3.90 (1.10–13.78) and AA vs GG p value = 0.063; OR = 3.24(0.94–11.19). (Table 5). No association was found in women (AA vs GG p value = 0.244; OR = 1.70 (0.70–4.17); AA vs AG p value = 0.277; OR = 1.66(0.67–4.12); AG vs GG p value = 0.883; OR = 1.03(0.73–1.43)) (Table 5).

**Table 4:** Association between rs939347 and the risk of obesity.

	Non-obese	Obese
***AA*** ** (n = 51)**	10 (20%)	41 (80%)
***AG*** ** (n = 384)**	144 (38%)	240 (62%)
***GG*** ** (n = 751)**	260 (35%)	491 (65%)
**OR (95%CI)** **AA vs AG**	2.46 (1.19–5.06)*P* = 0.015	
**OR (95%CI)** **AA vs GG**	2.28 (1.12–4.64)*P* = 0.024	
**OR (95%CI)** **AG vs GG**	0.90 (0.69–1.16)*P* = 0.406	

Obesity risk is defined as BMI > 30 kg/m^2^. Abbreviations: CI, confidence interval; OR, odds ratio; SNP, single-nucleotide polymorphism. *P* values were adjusted for age and gender.

**Table 5:** Association between rs939347 and the risk of obesity by gender.

	Lean men	Obese men
***AA*** **(Total N = 51)** **Total men = 24 (47%)**	3 (12%)	21 (88%)
***AG*** **(Total N = 384)** **Total men = 157 (41%)**	60 (38%)	97 (62%)
***GG*** ** (Total N = 751) Total men = 293 (39%)**	92 (31%)	201 (69%)
**OR (95%CI) in men** **AA vs AG**	3.90 (1.10–13.78)*P* = 0.035	
**OR (95%CI) in women** **AA vs AG**	1.66 (0.67–4.12)*P* = 0.277	
**OR (95%CI) in men** **AA vs GG**	3.24(0.94–11.19)*P* = 0.063	
**OR (95%CI) in women** **AA vs GG**	1.70 (0.70–4.17)*P* = 0.244	
**OR (95%CI) in men** **AG vs GG**	0.73 (0.48–1.09)*P* = 0.126	
**OR (95%CI) in women** **AG vs GG**	1.03 (0.73–1.43)*P* = 0.883	

Obese is defined as having a BMI>30 kg/m^2^. Lean is defined as having a BMI<26 kg/m^2^. Abbreviations: CI, confidence interval; OR, odds ratio.

**Figure 1:**
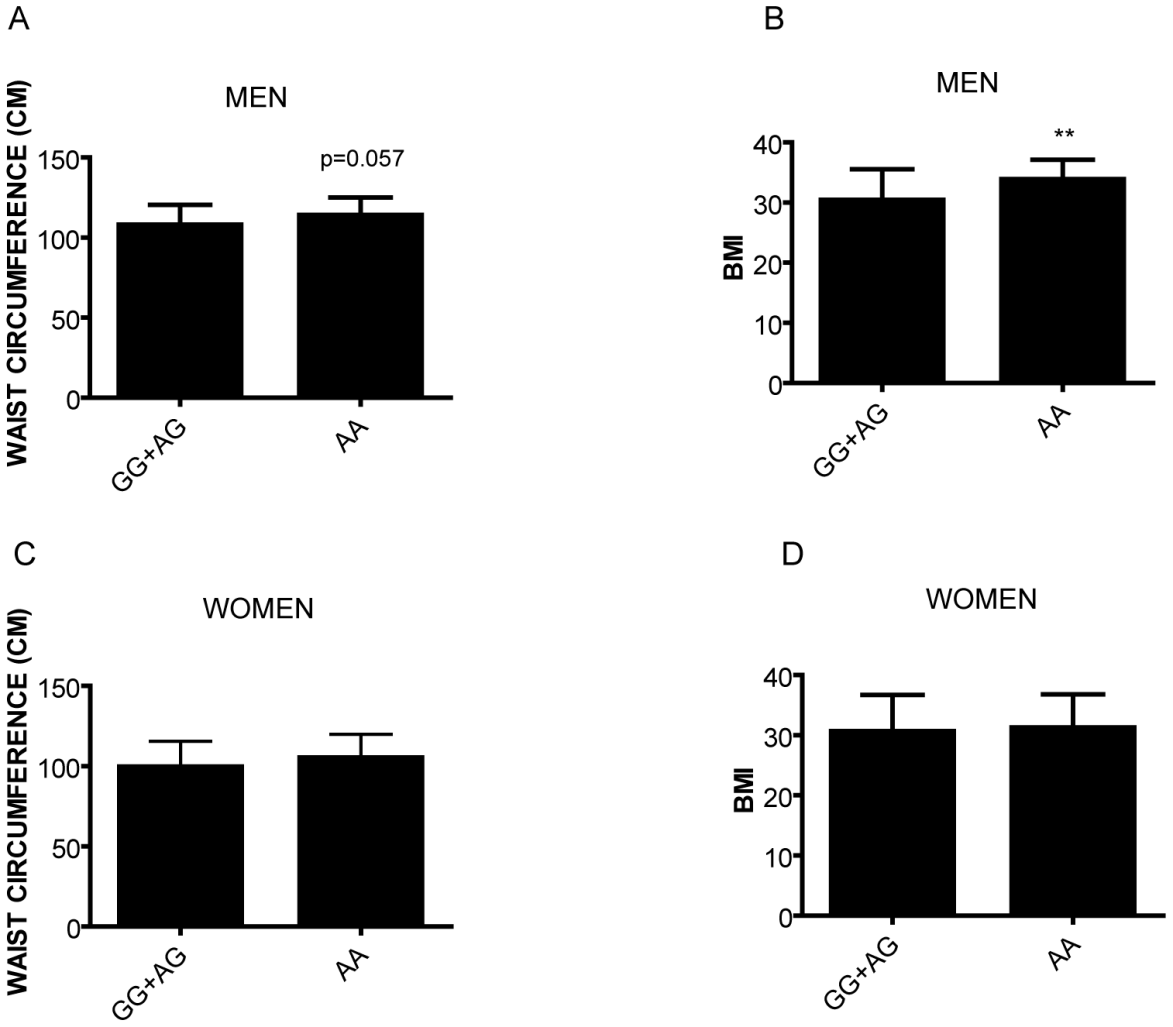
Association of rs939347 with obesity features. A) Waist circumference in men. B) BMI in men C) Waist circumference in women D) BMI in women.

In the total population, we observed that the AA-carriers showed higher BMI than AG+GG subjects (31.91±5.09 in AA vs 30.32±5.81 in AG+GG; t test p value = 0.04) (data not shown). When we divided the population by gender, men with AA-carriers showed a strong tendency (p = 0.057) to have higher waist circumference (113.4±11.6 cm vs 107.4±7 cm) (Figure 1A) and showed a statistically significant higher BMI (33.7±3.4 vs 30.3±5.3) (Figure 1B) as compared to the GG+AG group. In women we did not find any association between rs939347 and clinical parameters of obesity as waist circumference (Figure 1C) or BMI (Figure 1D). Furthermore, we did not find any association between the rs939347 and other clinical parameters in our cohort (data not shown). Haplotype analysis performed with Thesias 3.1 did not show any haplotype associated with obesity and type 2 diabetes. Thus, our data demonstrated novel associations in *REV-ERB ALPHA* between rs939347 and obesity traits in men.

### Luciferase assay study

Since rs939347 is located in the promoter region of Rev-erb alpha, we hypothesized that this SNP could alter the expression of Rev-erb alpha. To address this question, we performed a luciferase assay in two different cell lines: the human fibroblast HEK293, and the mouse preadipocytes 3T3-L1. The assay showed that the presence of rs939347 did not alter the expression of firefly luciferase in neither HEK293 (Figure S1A) nor 3T3-L1 (Figure S1B). However, we cannot discard the possibility that this SNP could modify the expression of REV-ERB ALPHA under in other conditions and at different time points. The data was normalized with the levels of the wild type promoter.

## Discussion

The interest in the role of REV-ERB ALPHA in the development of obesity has risen during the last few years, not only for its role in the adipogenesis but also as an integrator of circadian rhythms and metabolism. However, the impact of genetic variants of *REV-ERB ALPHA* has not extensively been studied. To date, there are only two studies associating SNPs in the intron region of the *REV-ERB ALPHA* gene with BMI in adolescents and adults [10] and with low physical activity and obesity in the Mediterranean and North America populations [11]. Here, for the first time we identified the SNP rs939347 in the *REV-ERB ALPHA* promoter that was associated with obesity in the Spanish population. The SNP rs939347 is located in the *REV-ERB ALPHA* promoter in the position −346 of the initial ATG and to 280 bp from DR2 element which has been previously associated with bipolar disorder within the haplotype with rs12941497 [29].

We have performed a cross-sectional study using samples from two nation-wide biobanks in Spain, the Hospital Clinic-IDIBAPS (Barcelona) and the National DNA Bank (Salamanca), involving a wide representation of the Spanish population older than 50 years of age. Our cohort, showed the classical features of obesity and type 2 diabetes and, interestingly, lower levels of cortisol and melatonin. Both hormones are usually studied as a measurement of the circadian state due to their different peaks throughout the day [30]. Higher levels of cortisol have been related to obesity and central obesity conditions such as Cushing syndrome [31], however some studies have reported an inverse correlation between BMI and cortisol levels [32], [33], similar to what we observed in our population. A higher clearance of cortisol due to obesity as well as a deregulation of the circadian secretion of cortisol with higher levels at night and lower levels in the morning [33] are possible explanations for our findings. Likewise, melatonin has been found in higher levels in lean subjects. Melatonin is synthesized in the pineal gland and not only regulates the circadian and seasonal rhythms but also has antioxidant effects and is implicated in the control of glucose and lipid metabolism [34], [35]. Previous studies showed higher levels of nocturnal melatonin in obese than in BMI-matched type 2 diabetes and lean groups, without any differences among groups at other moments during the day [36]. Moreover, melatonin is negatively correlated with features of metabolic syndrome in obese women [30], indicating that changes not only in the amplitude but also in the circadian secretion of melatonin could be associated with alterations in metabolism found in obesity and insulin resistance. Although a single point measure is not indicative of the complete daily secretion of both cortisol and melatonin, our results nevertheless support previous work relating obesity and type 2 diabetes with chronodisruption.

On another note, we found that the AA-carriers including all obese subjects exhibited a 2.46 fold-risk of developing obesity as compared to the AG-carriers and a 2.28 fold-risk as compared to the GG-carriers. Moreover, the AA-carriers exhibited a higher BMI and waist circumference than their non-carriers counterparts, demonstrating that the presence of the AA genotype is correlated with clinical traits of obesity. Furthermore, the association between rs939347 and obesity is stronger in men, as we observed a higher prevalence of AA in obese men (88%) with OR associated with 3.9-fold risk in AA carriers as compared to AG-carriers. In addition, male carriers of the AA-genotype have a higher BMI and waist circumference, suggesting an important role of rs939347 as a predictor of obesity in men. The differences in fat distribution and in physiological and behavioural components between men and women are evident, as well as other precedents of obesity risk factors associated with gender. For instance, the Pro12Ala polymorphism (rs1805192) in *PPARG* has been found associated with obesity in non-diabetic men [37]. Conversely, the 3111T/C (rs1801260) in *CLOCK* protect against obesity and overweight only in women [8]. Since REV-ERB ALPHA is implicated in adipogenesis, the presence of this SNP could favour a visceral accumulation of fat, especially in men. Thus, our results suggest that REV-ERB ALPHA could play a differential role in adipogenesis between men and women.

Luciferase assay did not show any statistical significance in the effect of rs939347 on the expression of firefly luciferase in the two cells lines studied. Although we did not find alterations in the expression of REV-ERB ALPHA with the AA genotype in 3T3-L1 preadipocytes, we can not discard the possibility that this SNP exerts a function during differentiation to adipocytes, as the mRNA levels of REV-ERB ALPHA increase during the differentiation process [38]. Likewise, further studies are necessary to determine if the presence of AA genotype alter the circadian expression of REV-ERB ALPHA in adipose tissue. Finally, in our study we did not find any association between rs2071427 and BMI. It was reported that rs2071427 is associated with body fat mass in a French population [10]. However, in the present study we could not find any association of rs2071427 with BMI. One of the reasons could be the different types of cohorts used in both studies and we cannot discard the impact of rs2071427 on obesity phenotypes.

REV-ERB ALPHA is a nuclear receptor located in chromosome 17q11.2 and is transcribed from the opposite strand of the *THRA* gene [39]. REV-ERB ALPHA mainly inhibits transcription in association with co-repressor NCoR [40] and chromatin remodelling in the element response in the target genes through the recruitment of HDAC3 [41]. Studies performed in the knockout mouse models of REV-ERB ALPHA showed a preference for lipid utilization instead of glucose, especially during the resting period, in addition to higher levels of glucose due to the misalignment of energy utilization, as well as higher adipose accumulation following a high fat diet [42]. These findings point to the important role of REV-ERB ALPHA in the control of glucose and lipid metabolism. In addition, it actively participates during the differentiation from preadipocytes to adipocytes along with PPARγ [23]. Therefore, changes in its expression or disturbances in its regulation due to the presence of genetic variations could be associated with modifications in adipogenesis as well as with misalignments in the circadian expression of metabolic genes that could lead to increase in adiposity and incidence of obesity. We can speculate that the higher frequency of AA in the obese group and its association with higher BMI and waist circumference could alter the expression or the regulation of REV-ERB ALPHA and its target genes. Alternatively, rs939347 could be in linkage disequilibrium with other functional SNPs in the Rev-Erb alpha gene that could affect its regulation. Future studies should be done to investigate this hypothesis.

The main limitation of our study is the use of a unique cohort to test SNP rs939347, although our population is not subjected to stratification, as all participants were from Spain. However, it is essential to replicate our results in other populations. Previously, Goumidi et al. [10], did not find any association of rs2071570 (which is tagging of rs939347) with BMI. Nevertheless, as we mentioned before, either the types of cohorts studied or the statistical approximation used differed. On the other hand, we did not obtain any information about sleep duration, or time of the day (morning vs evening) that could associate this polymorphism with a disruption in the sleep cycles.

In conclusion, we found a new SNP in *REV-ERB ALPHA* promoter, rs939347 which is associated with obesity in the Spanish obese male population. This work supports the role of REV-ERB ALPHA in the development of obesity and as a potential target in the treatment of obesity.

## Supporting Information

Figure S1 **Luciferase assay of rs939347.** A) Luciferase assay of rs939347 performed in HEK293 B) Luciferase assay of rs939347 in 3T3-L1.
(TIF)

Table S1 **List of primers used to amplify the promoter of REV-ERB ALPHA.**
(DOC)

Table S2 **SNPs found in the promoter of REV-ERB ALPHA gene.**
(DOC)
