# Supplementary figures and images for: REV-ERB ALPHA Polymorphism Is Associated with Obesity in the Spanish Obese Male Population
Source: PLoS One. 2014 Aug 4;9(8):e104065. doi: 10.1371/journal.pone.0104065 (PMC4121274; doi:10.1371/journal.pone.0104065)

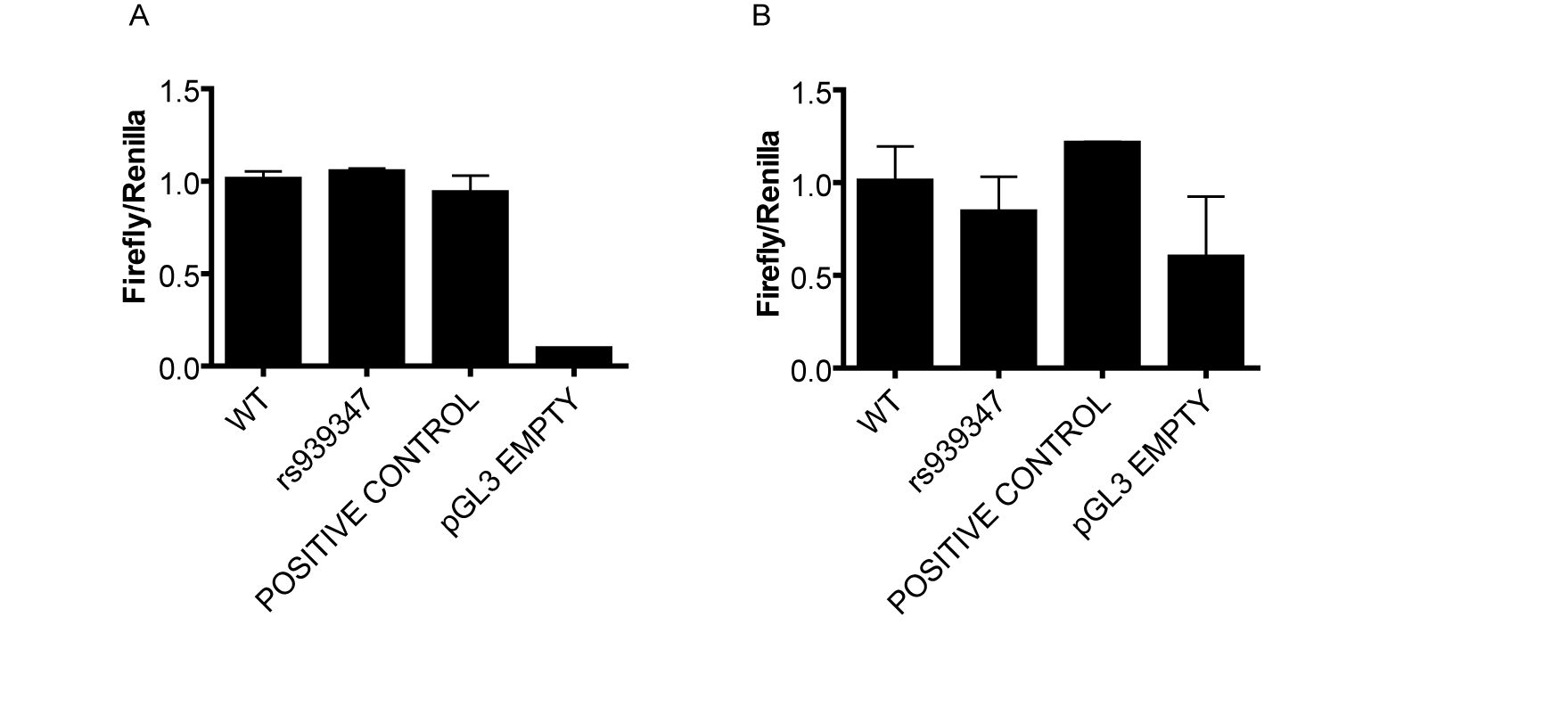

Supplement: Figure S1 — Luciferase assay of rs939347. A) Luciferase assay of rs939347 performed in HEK293 B) Luciferase assay of rs939347 in 3T3-L1. (TIF) [file pone.0104065.s001.tif]
