# Supplementary material for: REV-ERB ALPHA Polymorphism Is Associated with Obesity in the Spanish Obese Male Population
Source: PLoS One. 2014 Aug 4;9(8):e104065. doi: 10.1371/journal.pone.0104065 (PMC4121274; doi:10.1371/journal.pone.0104065)
Supplement: Table S1 — List of primers used to amplify the promoter of REV-ERB ALPHA. (DOC) [file pone.0104065.s002.doc]

**Table S1.**

List of primers used to amplify the promoter of REV-ERB ALPHA.

| ***Primers*** | ***Sense*** | ***Antisense*** |
| --- | --- | --- |
| REV-ERB ALPHA 1A | ctctggactttgccctgaag | gctgtctgggagatcaaagc |
| REV-ERB ALPHA 1B | CACTCTCAGCTCCTCCCA | TGCCAATCTCAGCCGCCTT |
| REV-ERB ALPHA 1C | AGGGAAAGGCTCGGGCAAA | GCATGGGAAGAGCAGAGAGA |
| REV-ERB ALPHA 1D | TCTCTCTGCTCTTCCCATGC | GACACCCCAGTCCCTTACAA |
