# Supplementary material for: REV-ERB ALPHA Polymorphism Is Associated with Obesity in the Spanish Obese Male Population
Source: PLoS One. 2014 Aug 4;9(8):e104065. doi: 10.1371/journal.pone.0104065 (PMC4121274; doi:10.1371/journal.pone.0104065)
Supplement: Table S2 — SNPs found in the promoter of REV-ERB ALPHA gene. (DOC) [file pone.0104065.s003.doc]

**Table S2.**

SNPs found in the promoter of REV-ERB ALPHA gene

| ***SNP*** | ***Nucleotide change*** | ***Position*** | ***MAF*** |
| --- | --- | --- | --- |
| rs2071570 | C>A | -743 | 0.3 |
| rs143309510 | C>T | -677 | 0.08 |
| rs199703673 | C>T | -459 | 0.002 |
| rs939347 | G>A | -346 | 0.36 |
| rs183213308 | G>C | -363 | 0.01 |
| rs17616365 | C>T | -54 | 0.01 |
